# Unilateral Type of Macrodystrophia Lipomatosa of the Thumb, Index Finger, and Thenar

**DOI:** 10.4274/balkanmedj.2018.1322

**Published:** 2019-01-01

**Authors:** Ivana Kavecan, Milan Obrenovic, Boris Privrodski, Djerdji Erdes Kavecan, Zoran Golusin

**Affiliations:** 1 University of Novi Sad Faculty of Medicine, Hajduk Veljkova, Novi Sad, Serbia; 2 Institute for Children and Youth Health Care of Vojvodina, Hajduk Veljkova, Novi Sad, Serbia; 3 Department of Philosophical Scienses, Study group Psychology, State University of Novi Pazar, Novi Pazar, Serbia; 4 Department of Dermatology, Clinical Centre of Vojvodina, Hajduk Veljkova, Novi Sad, Serbia

Macrodystrophia lipomatosa, also known as type 1 macrodactyly or isolated macrodactyly, is a rare, benign, congenital, nonhereditary condition, generally unilateral, involving one or more fingers. Affected body parts manifest overgrowth in length and girth due to proliferation of fibroadipose tissue but only until puberty when it usually ceases. It occurs in approximately 1 in 100,000 live births (1,2).

Herein, we describe the case of a 19-month-old boy with localized enlargement of the thumb, index finger, and thenar of the right hand observed at birth that gradually progressed over time (Figure 1a, 1b). He had difficulty in flexion of the interphalangeal joint of the thumb but showed normal flexion of the metacarpophalangeal joint of the thumb. Other fingers of the same hand (III, IV, and V) and all digits of the left hand and feet were normal. Other body proportions were normal without any other dysmorphological features. No additional abnormalities of the limbs or other organ systems were observed. The patient’s parents were not concerned about these abnormalities and had sought advice for purely cosmetic reasons. Radiograph of the affected fingers showed soft tissue and bony overgrowth in the metacarpal bones of the thumb and index finger. The second metacarpal bone presented an arched deformity. There was medial luxation of the interphalangeal joint of the index finger, and the medial phalange was disproportionately smaller (Figure 1c). Magnetic resonance imaging of the right hand revealed enlargement of the first metacarpal bone and both index phalanges and enlargement of subcutaneous tissue of the thumb, index finger, and thenar (Figure 1d). Written informed consent was obtained from the patient’s parents for the publication of this report.

Localized overgrowth syndromes such as macrodystrophia lipomatosa have been recognized as a part of overgrowth spectrum disorders related to phosphatidylinositol-4,5-bisphosphate 3-kinase catalytic subunit alpha (PIK3CA) whose genotype-phenotype correlation has not been sufficiently understood (3,4). In this report, the patient’s mother did not permit further invasive procedures such as biopsy or surgical debulking, and hence, we could not identify the exact mutation causing macrodystrophia lipomatosa in this patient. Nevertheless, it is not always possible to detect mutations, even in cases where they exist, due to low-level somatic mosaicism (3), and these patients should be monitored for other possible associated complications such as vascular malformations and skeletal abnormalities (5).

In conclusion, we report a rare and interesting case of macrodystrophia lipomatosa as a part of PIK3CA-related overgrowth spectrum disorders. However, clinicians should recognize the necessity for further evaluation of somatic mutations in the differential diagnosis of macrodystrophia lipomatosa. When parents of pediatric patients do not permit further diagnostic investigation or intervention, as observed in our case, the only possibility of establishing a diagnosis is through evaluation of clinical features and imaging findings.

## Figures and Tables

**Figure 1:**
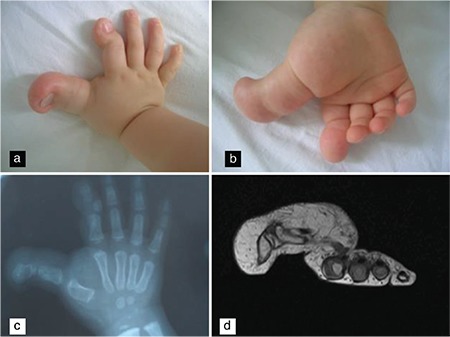
Affected fingers and thenar of the right hand showing enlargement (a, b). Radiograph of the affected fingers showing soft tissue and bony overgrowth in the metacarpal bones of the thumb and index finger (c). Magnetic resonance imaging showing accumulation of fatty tissue (d).
